# Protocol for a murine skin abscess model to study bacterial infection dynamics, microbial interactions, and treatment efficacy

**DOI:** 10.1016/j.xpro.2025.104291

**Published:** 2025-12-20

**Authors:** Anupriya Gupta, Kathleen J. Sircombe, Janaya D. Stevenson, Samuel J.T. Wardell, Daniel Pletzer

**Affiliations:** 1 Department of Microbiology and Immunology, Faculty of Biomedical Sciences, University of Otago, Dunedin, New Zealand

**Keywords:** health sciences, immunology, microbiology, model organisms

## Abstract

We present a murine skin abscess model to study pathogen-pathogen and host-pathogen interactions, infection progression, and therapeutic interventions. We outline steps for real-time monitoring of bacterial infection dynamics and host responses using bioluminescent or fluorescently labeled bacteria using IVIS (*in vivo* imaging system). We provide details to isolate bacterial and host RNA/DNA from infected tissues, enabling downstream transcriptomic and molecular analyses. This model offers a robust platform to study bacterial pathogenesis and host immunity and evaluate antimicrobial or innovative treatment strategies.

For complete details on the use and execution of this protocol, please refer to Pletzer et al.^1^

## Highlights

• Procedures for establishing chronic subcutaneous abscesses using WHO-priority pathogens
• Steps for infecting mice with pathogens and harvesting abscess tissue
• Instructions for *in vivo* live imaging of infection
• Guidance on administering antibiotics intra-abscessly and quantifying treatment effects

## Abstract (editor highlights)

Publisher’s note: Undertaking any experimental protocol requires adherence to local institutional guidelines for laboratory safety and ethics.

## Graphical abstract

**Figure:**
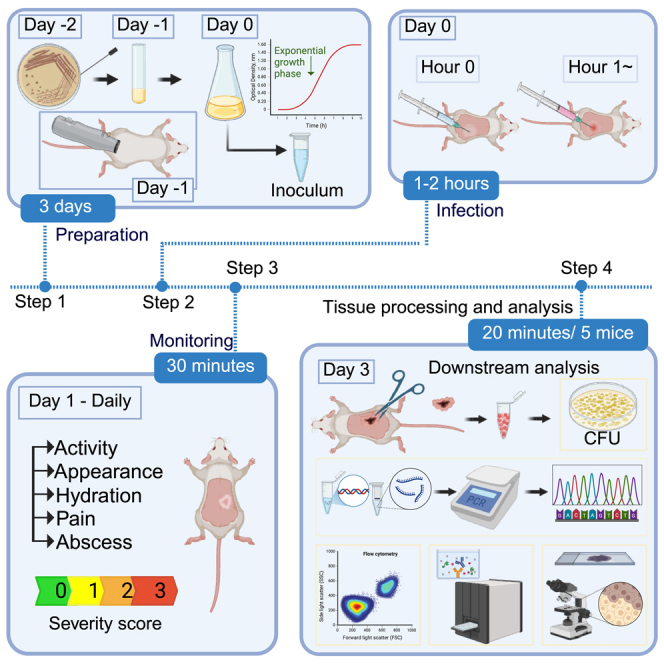

## Before you begin

This protocol outlines a murine skin abscess model using a high-density bacterial inoculum to study infection progression and evaluate antimicrobial treatments. Immunocompetent mice typically develop a localized skin infection within a standardized 3-day experimental window (Figure S1) when established bacterial pathogens are used. Kaplan–Meier curves in Figure S1 show 100% survival across multiple bacterial strains and inoculum doses. New organisms should first be assessed for their ability to induce infection.

Bacterial infections can be tracked non-invasively using bioluminescent or fluorescently labelled bacteria.^1^ This protocol uses clinical, multidrug-resistant strains *Pseudomonas aeruginosa* LESB58 and *Staphylococcus aureus* LAC USA300. However, the model is broadly compatible with other well-characterized pathogens^1^^,^^2^ and suitable for co-infections.^3^^,^^4^ Bacterial concentration should be optimized to establish robust infection while avoiding levels that compromise mouse welfare. Before testing therapeutic compounds, toxicity screening is required. We have assessed diverse compounds, including peptides,^5^ peptidomimetics,^6^ nanogels,^7^ liposomes,^8^ nanoparticles,^9^ and antibiotic combinations.^10^

Swiss Webster (SW) mice were used, though CD-1 or C57BL/6 are also suitable. Housing does not require specific pathogen-free conditions. A minimum three-day acclimatization is recommended. All infections and handling should occur in a Biosafety Level 2 (BSL-2) laboratory under appropriate containment conditions.

Basic proficiency in handling laboratory mice is essential, although the required manipulations are relatively straightforward. Depending on the planned downstream processes, proficiency in data acquisition and interpretation is necessary.

### Innovation

This protocol presents a streamlined, highly reproducible murine model that enables robust investigation of chronic, high-density Gram-positive and Gram-negative bacterial infections. Unlike existing models that rely on technically demanding procedures, such as thermal injury, surgical implantation, immune suppression, or embedding bacteria in agarose or biofilm matrices, this model establishes long-lasting cutaneous abscesses through a simple subcutaneous injection underneath the panniculus carnosus. The model supports persistent, non-disseminating infections for up to 10 days without requiring mouse manipulation or specialized inoculum preparation.

A key advancement is the use of the multidrug-resistant *P. aeruginosa* cystic fibrosis epidemic isolate LESB58, whose reduced motility enables stable, localized abscess formation. This strain allows for consistent, high-density infections that can be longitudinally monitored in live animals using lux-tagged or fluorescently labelled bacteria, fluorescent immune cell trackers, and ROS/RNS imaging. The model thereby integrates noninvasive tracking of bacterial proliferation, host inflammatory responses, and disease progression within a single workflow.

The protocol further extends to multiple clinically relevant, drug-resistant bacteria, including WHO-priority pathogens *Acinetobacter baumannii*, *Klebsiella pneumoniae*, *Enterobacter cloacae*, *Enterococcus faecalis,* and *Escherichia coli*, demonstrating its broad applicability. It also supports virulence assessments using mutant strains and allows evaluation of antimicrobial efficacy under physiologically relevant, high-density infection conditions, where conventional systemic therapy is often ineffective.

Overall, this model provides an accessible, scalable platform that merges simplified animal handling, real-time imaging, and high reproducibility. This is a significant advancement over traditional chronic infection models for studying pathogen-pathogen and host–pathogen interactions and testing anti-infective strategies.

### Institutional permissions

Mice used in this study were female Swiss Webster aged approximately 6–7 weeks and sourced from the University of Otago Biomedical Research Facility. All animal experiments were approved by the University of Otago Animal Ethics Committee under protocol number AUP19-125.

### Timeline

**Figure 1:**
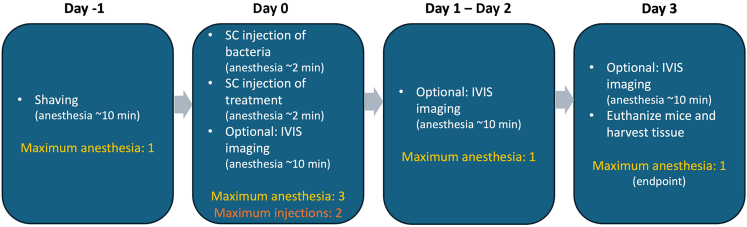
Timeline of a standard experimental setup Illustrates the maximum duration of anesthesia sessions and the number of injections required.

Procedural steps for a murine subcutaneous infection model, including optional imaging, anesthesia session, and tissue collection (Figure 1).

***Note:*** This timeline reflects a standard experimental setup in which mice are euthanized 3 days post-infection. However, the duration between infection and experimental endpoint may vary, depending on the bacterial strain used and the specific objectives of the study.

#### Day −2

1. Streak the selected bacterial strain onto an appropriate nutrient-rich agar plate.
2. Confirm that all mice are healthy, well-acclimatized, and co-housed without signs of aggression.

#### Day −1

3. Shave the dorsal area of each mouse to expose skin.
4. Inoculate a single bacterial colony into liquid broth and incubate overnight shaking at the appropriate temperature.

#### Day 0

5. Sub-culture the overnight culture to the mid-log phase and adjust to the desired inoculum concentration.
6. Prepare treatments and vehicle controls.
7. Infect mice subcutaneously with the bacterial suspension and administer treatments as required.

#### Day 1 and 2

8. Monitor mice visually and/or IVIS to assess infection progression. Monitor for clinical signs, abscess development, and welfare.

#### Day 3

9. Euthanize mice following approved ethical protocols.
10. Photograph abscesses, measure lesion size, and dissect the infected area.
11. Homogenize tissue for CFU enumeration and prepare samples for additional downstream analysis.

#### Day 4

12. Count CFUs and proceed with downstream analysis.

## Key resources table

**Table:** 

REAGENT or RESOURCE	SOURCE	IDENTIFIER
**Bacterial strains**		

*Staphylococcus aureus* USA300 LAC	Centers for Disease Control and Prevention (CDC)^11^	–
*Pseudomonas aeruginosa* LESB58	Cheng et al.^12^	–

**Chemicals**		

Chemical Depilator	Nair	-
Tricin Eye Ointment	Jurox	-
Tryptone	Condalab	Cat# 1612.00
Yeast extract granulated	Condalab	Cat# 1702.00
Agar powder for microbiology	NeoFroxx	CAS# 9002-18-0
Sodium chloride	Supelco	CAS# 7647-14-5
1**×** phosphate-buffered saline (DPBS) Tablets	Gibco	#18912-014

**Critical commercial assays**		

Pierce Rapid Gel Clot Endotoxin Assay Kit	Thermo Scientific	A43879
Pierce BCA Protein Assay Kit	Thermo Scientific	23225

**Experimental models: Organisms/strains**		

*P. aeruginosa* LESB58 pUCP.eGFP	Pletzer et al.^13^	–
*S. aureus* LAC USA300 pKK22.eqFP650	Wu et al.^14^	–
Mouse strain: Swiss Webster	University of Otago Biomedical Research Facility	*Mus musculus*, outbred, male or female, 6–7 weeks old
Mouse strain: C57BL/6 (BL-6)	University of Otago Biomedical Research Facility	*Mus musculus*, inbred, male or female, 6–7 weeks old

**Software and algorithms**		

Living image	PerkinElmer	–

**Other**		

Syringe 1 mL	BD	302100
96-well plate	Thermo Scientific (Nunclon Delta Surface)	Cat#167008
Conical tubes 15 mL, 50 mL	15 mL: Tarsons 50 mL: Cellstar Greiner Bio-One	15ml: Cat#546021 50ml: Cat#227-261
Surgical scissors	Copper Medical Limited	–
Digital Calliper	Marathon	CO030150
SilhouetteStar (3D wound camera)	Aranz	–
Clippers	Codos	–
Ceramic Beads, 2.8 mm (325 g)	QIAGEN	13114–325
Tissue lyser (Mini-beadbeater)	Mini G 1600, SPEX Sample Prep	–
Tissuelyser III	Qiagen	9003240
Centrifuge (big and small)	Big: Thermo Scientific (Heraeus Multifuge x1R Centrifuge), Small: Eppendorf Centrifuge 5425	Big: Ser#: 41701261 Cat#: 75004250 Small: SN54055JH415632

## Step-by-step method details

### Animal/bacteria preparation and shaving procedure

**Timing: 3 days**

This section prepares the bacterial inoculum and experimental animals to ensure reproducible infection outcomes. Bacteria are grown to mid-log phase and washed to remove residual growth media, endotoxins and proteins, to minimize unintended inflammation. Mice are shaved and depilated one day before infection to standardize skin condition and reduce variability in lesion development.

**Table 1:** Standard isoflurane dosing for mice (Inhalational anesthesia)

Phase	Isoflurane % in oxygen	Notes
Induction	3–5%	Fast induction, usually 30 s to 1 min
Maintenance	1–2.5% (can go up to 3%)	Lower concentration used for short-term imaging or surgery
Recovery	0%	Stop isoflurane and provide oxygen till normal movement resumes

**Table 2:** Inoculum used for subcutaneous abscess model

Strains	OD_600_ adjustment after wash	CFU correlation (CFU/mL)	Target inoculum (CFU/mouse)	Target inoculum range (CFU/mouse)
*Pseudomonas aeruginosa* LESB58	2	1 × 10^9^	5 × 10^7^	2 × 10^7^ - 1 × 10^8^
*Pseudomonas aeruginosa* PAO1	0.5	5 × 10^8^	2.5 × 10^7^	1 × 10^7^ - 5 × 10^7^
*Pseudomonas aeruginosa* PA14	0.1	1 × 10^8^	5 × 10^6^	2 × 10^6^ - 1 × 10^7^
*Staphylococcus aureus* LAC USA300	2	5 × 10^8^	2.5 × 10^7^	1 × 10^7^ - 5 × 10^7^
*Acinetobacter baumannii* Ab5075	100	2 × 10^10^	1 × 10^9^	5 × 10^8^ - 2 × 10^9^
*Klebsiella pneumoniae* KPLN649	100	2 × 10^10^	1 × 10^9^	5 × 10^8^ - 2 × 10^9^
*Enterococcus faecium* #1-1	100	2 × 10^10^	1 × 10^9^	5 × 10^8^ - 2 × 10^9^
*Enterobacter cloacae* 218R1	20	5 × 10^9^	2.5 × 10^8^	1 × 10^8^ - 5 × 10^8^
*Escherichia coli* E38	50	5 × 10^10^	2.5 × 10^9^	1 × 10^9^ - 5 × 10^9^
*Escherichia coli* MG1655	50	1 × 10^10^	5 × 10^8^	2.5 × 10^8^ - 1 × 10^9^

Bacteria were grown to an OD_600_ of 1.0 and washed twice in PBS before adjustment. Injection volume was 50 μL.

**Figure 2:**
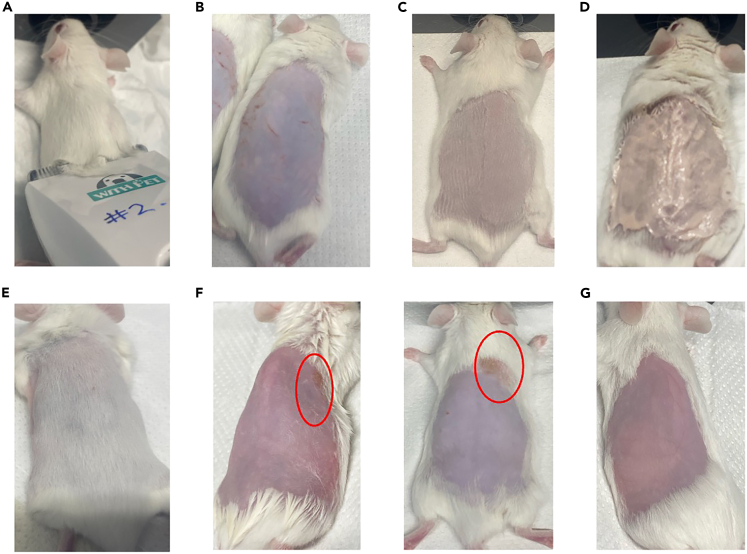
Mouse hair removal techniques and associated outcomes (A) Clipper positioning while shaving. (B) Skin abrasions resulting from improper use of hair clippers during trimming. (C) Close hair trimming. (D) Depilatory cream application. (E) Rapid hair regrowth observed in a young mouse (<5 weeks old) within 3 days post-improper shaving. (F) Burn marks on the skin caused by residual depilatory cream left on too long or not cleaned properly. (G) Skin appearance after complete hair removal using depilatory cream.

#### Day −2: Bacterial streaking and mouse shaving

1. Streak the selected bacterial strain onto a nutrient-rich (e.g., LB) agar plate.

***Note:*** Incubate overnight at 37°C for 12–16 h, or until well-defined colonies are visible.

### Day-1

2. Shave the mice on Day -1. Use clippers followed by hair removal cream to expose the dorsal skin area designated for infection.
  ***Note:*** We recommend shaving mice 1 day before infection to allow the skin to recover from any potential irritation or minor injuries caused by the hair removal process, particularly when using depilatory creams. Performing shaving and infection on the same day is possible; however, it may increase the risk of skin inflammation or micro-abrasions that could confound experimental outcomes by mimicking or amplifying infection-associated inflammation. Allowing a 24-h recovery period also helps ensure a more consistent baseline skin condition across animals, improving reproducibility and reducing variability in lesion development.
  a. Anesthesia.
    i. Mice should be anesthetized using isoflurane (3%–5% isoflurane, 2% isoflurane for maintenance). Isoflurane usage conditions, including induction and maintenance doses, are summarized in Table 1.
    ii. Place mice on a heating pad to prevent hypothermia.
    iii. Confirm anesthesia by pinching the foot; mice should not flinch.
      ***Note:*** Additional anesthesia during shaving or infection is justified to prevent burns or movement that can confound results.
    iv. Apply sterile ophthalmic lubricant (Tricin, Jurox) to prevent eye dryness.
  b. Hair removal.
    i. Lay the anesthetized mouse flat on the heated mat, back facing up, with nose in nose cone for continued anesthesia.
    ii. Use electric clippers (Codos) to remove fur from the back of the mouse. Begin shaving at the hind legs, moving against the direction of fur growth up to the thoracic region.
    iii. For best results, hold the clippers at a 30° angle to the mouse’s body, and move in smooth, long strokes toward the head (Figure 2A) and at a flat angle to prevent nicks (Figure 2B) or uneven hair removal.
      ***Note:*** A close shave (Figure 2C) ensures that minimal hair-removal cream is needed, reducing burns. Also, depilatory creams are ineffective on dense, long fur. Ensure that the back area is shaved thoroughly before adding the cream.
  c. Application of chemical depilatories and removal.
    i. Prepare a beaker of lukewarm tap water.
    ii. Apply a sufficient, even layer of chemical depilatory (e.g., Nair) to the shaved area using a cotton swab or gauze. Leave the depilatory for one minute, depending on the product used (Figure 2D).
      ***Note:*** Small amounts of depilatory cream can be reapplied to areas with residual hair, if necessary. Precise timing is critical insufficient application (too short) may leave patches of fur or lead to rapid regrowth, while prolonged exposure can cause chemical burns and skin irritation. Depending on the product used, we recommend starting with a 60-s application time, followed by gentle removal and assessment. Adjust the exposure duration as needed based on mouse strain-specific skin sensitivity and product performance.
      **CRITICAL:** Hair removal timing is critical for consistency. Insufficient exposure to depilatory cream may leave residual hair (Figure 2E) and interfere with infection readouts, while prolonged exposure can cause chemical burns (Figure 2F) or skin irritation. If hair removal is incomplete, rinse the depilatory thoroughly and reapply for one additional minute only.
    iii. Use pre-cut 5×5 cm dry gauze to remove most of the depilatory cream gently.
      ***Note:*** Do not use paper towels as they can irritate the skin.
    iv. Dampen gauze with lukewarm water to remove any remaining depilatory cream thoroughly.
    v. Start at the bottom of the shaved area and move toward the head, against the direction of hair growth.
    vi. Dry the skin using fresh, dry gauze to prevent irritation or burns. Keep the mouse on the heating pad to ensure its body temperature does not drop.
    vii. Ensure that all depilatory has been thoroughly removed from the mouse, paying extra attention to the boundary between the shaved and fur-covered area (Figure 2G).
      **CRITICAL:** Any remaining cream will result in burns (Figure 2F), which is an ethical concern and may interfere with abscess development, including undesirable immune response.
      ***Note:*** Male and female mice can be mixed in the study; however, males tend to fight more in group housing. If you observe excessive scratches or injuries, separate them. Male mice of the same age as females have thicker skin, often resulting in smaller abscesses.
      ***Note:*** We have tested Swiss Webster, CD-1, and C57BL/6 (BL-6) mice, all of which work well. Note that BL-6 mice of the same age are smaller in size, and their wounds may appear more severe. However, they generally tolerate infections well. C57BL/6 mouse skin is multicolored, which can result in difficulty in identifying hair removal and abscess development.
      ***Note:* Post-depilation care:** After removing the depilatory cream with lukewarm gauze, check carefully for any residual cream trapped in the fur around the shaved area. A pink-colored depilatory cream is easier to detect than a white cream. Even small amounts of residual cream can cause severe burns, leading to exclusion from experimental studies. If shaving and injections are performed on the same day, residual depilatory cream can be easily overlooked, which could cause unfavorable experimental outcomes. Damage to the skin before infection is likely to result in upregulation of the immune response in the skin, which could alter results.
      ***Note:* Ethical considerations:** Ethics committees may raise concerns about the use of additional isoflurane for shaving and infections; however, this can be justified by explaining the risk of burn wounds, as burns may not be visible for a few hours. We have also observed that when bacterial suspensions are left in buffer for extended periods, abscess formation becomes more variable and less reproducible. From a biosafety standpoint, it is also advisable to separate the infection procedure, which requires sterile technique, from other preparatory steps, such as shaving. This separation reduces the risk of cross-contamination.

3. Pick one colony from the agar plate and inoculate 5 mL of nutrient-rich medium using a 25 mL culture tube.

***Note:*** Prepare duplicates of your overnight culture.

4. Grow overnight shaking at 37°C for 12–16 h.

***Note:*** We recommend using a 1:5 liquid:air ratio and 250 rpm shaking at 37°C for most aerobic bacteria (i.e., 20 ml culture in a 100 ml flask).

#### Day 0: Bacterial inoculum preparation

5. From the overnight culture, adjust the bacterial culture to an OD of 0.1 using a spectrophotometer in a conical flask.

***Note:*** Perform growth curve analysis of all bacterial strains to determine the length of time bacterial cultures take to grow to mid-log phase (Day 0). This can range from 2 h (some *S. aureus* strains) to 4–6 h (slower-growing cultures, such as *P. aeruginosa* LESB58). The time the culture takes to mid-log phase should be used as an indicator of potential contamination (i.e., if cultures are growing too slowly or too fast, it could indicate contamination, and experiments should be terminated before going into animals). Moreover, inconsistency in culture preparation can result in altered gene expression (i.e., virulence) and lead to inconsistent abscess formation in control mice.

6. Incubate bacteria in a shaking incubator until the mid-log phase at 37 °C.

**CRITICAL:** Always grow microorganisms to mid-log phase to ensure reproducibility. Avoid using stationary-phase cultures or diluting overgrown cultures, as this can introduce variability.^15^^,^^16^ Consistently preparing cultures at the same growth stage will help maintain uniformity in abscesses (skin lesions).

7. Spin down bacterial culture at 5,364 g for 4 min at room temperature (18–22°C).
8. Remove the supernatant and resuspend the bacterial pellet in 1 mL sterile PBS; wash again by centrifuging at 5,364 g for 4 min at room temperature (18–22°C) in a microcentrifuge tube.
9. Decant the supernatant and resuspend the pellet in 1 mL of sterile PBS. Ensure the pellet is fully resuspended. Measure the absorbance (OD_600_) using a spectrophotometer to further adjust the inoculum concentration as needed.
10. Adjust cultures to the final OD appropriate for your respective strain per 50 μL inoculum, depending on the strain used (see CFU-OD correlation for commonly used strains that consistently produce reproducible abscesses and bacterial counts, provided in Table 2).

***Note:*** We use 50–100 μL of inoculum for SC injection. Care must be taken to ensure that sufficient bacteria are present in the volume selected.

11. Aliquot 500 μL of the final bacterial suspension into endotoxin-free reaction tubes (recommended at least once per new strain).
  a. Measure residual endotoxin using the Rapid Gel Clot Endotoxin Assay Kit (Pierce™ Rapid Gel Clot Endotoxin Assay Kit, A43879) according to the manufacturer’s instructions.

***Note:*** The kit has a detection limit of 0.03 EU mL^−1^.

12. Aliquot 100 μL of the same bacterial suspension into a separate tube for protein estimation.
  a. Measure residual protein using the Pierce BCA Protein Assay Kit (Thermo Scientific, 23225) according to the manufacturer’s instructions.

***Note:*** Protein levels must be ≤1 μg/mL to exclude medium-derived protein contamination that could induce inflammation.

**CRITICAL:** Verification of residual endotoxin and protein levels in the final bacterial suspensions is essential for ensuring reproducibility and avoiding artefactual inflammation. Both parameters must fall below the defined thresholds (≤0.03 EU mL^−1^ endotoxin and ≤1 μg mL^−1^ protein). Exceeding either threshold may confound lesion development and invalidate experimental data.

13. Set aside 20 μL of each final culture and serially dilute using a 96-well plate to determine CFUs of the inoculum.

***Note:*** Before infecting mice, test the relationship between OD and CFU for your bacterial strains to ensure you are infecting with an adequate concentration of bacteria to induce the infection.

***Note:*** If performing transcriptomic downstream analyses of bacteria after the experiment, set an aliquot aside for DNA/RNA extractions.

**CRITICAL:** Inoculum should not contain any growth media and should be resuspended in endotoxin-free sterile PBS, saline, or glucose. Avoid using pure water due to osmotic imbalance. It can also irritate tissue and trigger an inflammatory response, and in the worst-case scenario, cause necrosis.

***Note:*** This protocol can also be used to develop polymicrobial infections. Care must be taken when testing bacterial ODs in mice for polymicrobial experiments to ensure mice can tolerate the infection.^1^^,^^2^ We recommend preparing bacteria separately, adjusting the OD to the desired density, and then combining them immediately before injection in a desired volume ratio (e.g., 1:1).

### Subcutaneous infection and treatment

**Timing: 1–2 h**

This section describes the procedure for establishing a subcutaneous bacterial infection in mice and administering therapeutic interventions. It ensures precise injection and pre-testing of compounds for local and systemic toxicity, enabling reliable evaluation of treatment efficacy while maintaining animal welfare.

**Figure 3:**
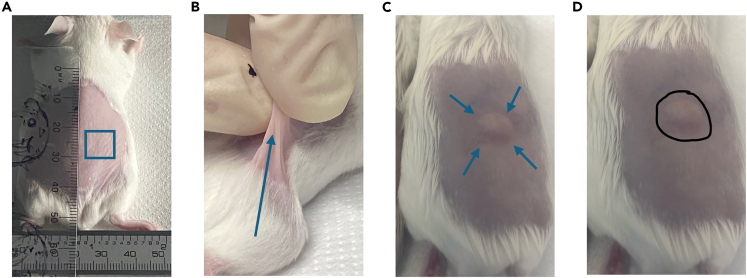
Subcutaneous injection technique in mice (A) Schematic representation of subcutaneous injection sites, approximately 1–2 cm posterior to the shoulder blades and 0.5–1 cm lateral to the midline. (B) Formation of a ‘tent’ by gently lifting the skin to facilitate subcutaneous injection. (C) Bleb at the sight of SC injection. (D) Marking the injection site using a permanent marker to track the location of inoculation.

**Figure 4:**
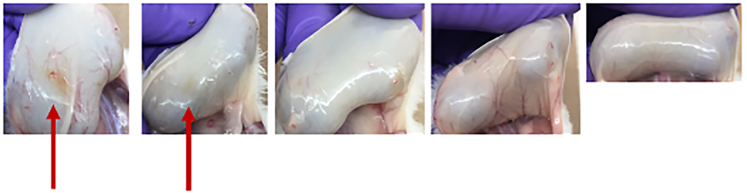
Maximum tolerated dose testing using various concentrations to identify the one that does not cause harm to use for efficacy tests

**Figure 5:**
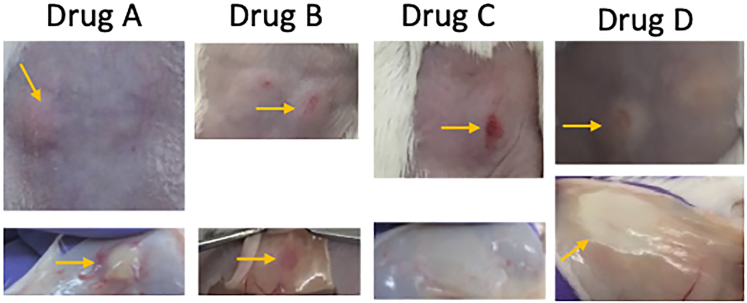
Skin toxicity screening of new compounds Drug A results in visible subcutaneous precipitation accompanied by pronounced inflammation in the overlying skin flap. Drug B induces surface-level skin irritation with extensive underlying inflammation. Drug C causes localized necrosis without visible subdermal precipitation. Drug D produces a similar necrotic profile but with more widespread subcutaneous aggregation and potential early-stage pus formation.

#### Day 0: Infection

14. Anaesthetize mice using isoflurane as described in step 2 (3%–5% of isoflurane, Table 1). Transfer the mouse onto the heating mat, place the mouse head into the nose cone with 2% isoflurane, and keep the mouse in a horizontal position.

***Note:*** Since the injection is rapid and minimally invasive, mice do not need to reach a surgical plane of anesthesia. However, we recommend brief immobilization of the mice to ensure accurate injection into the dorsal flank (as mentioned in step 16) and to allow sufficient time to mark the injection site. If a mouse does not regain consciousness promptly, it should be placed in a clean recovery cage lined with paper towels and maintained on a heat source. Ophthalmic lubricant should be applied to prevent corneal drying, and the animal must be continuously monitored until fully mobile.

***Note:*** To ensure consistency across experiments, it is recommended that each experimental batch include three control mice at the previously determined CFU. Data from these reference mice are used to monitor batch variability, and any experimental batch in which the reference CFU recovery deviates by more than one log_10_ from the historical mean should be carefully reviewed and, if necessary, excluded and the experiment repeated. We also recommend performing each experiment at least three times using an independently prepared bacterial inoculum and mice from different litters. Experiments should not rely on a single cohort of mice.

15. Vortex the inoculum to ensure a homogeneous bacterial suspension. Draw up 50 μL of suspension into the 29-Gauge needle. Ensure that no bubbles are present at the plunger/syringe interface.

***Note:*** Syringes with detachable needles are not recommended because the dead space between the syringe barrel and the needle hub can lead to variability in the injected volume. This is particularly critical when working with bacterial suspensions, where even slight differences in volume can alter the bacterial load.

16. Inject 50 μL of bacterial suspension subcutaneously.
  a. Inject under the thick skeletal muscle/panniculus carnosus at the either side of the shaved dorsal flank of the mouse (∼1–2 cm posterior to the shoulder blades and ∼0.5–1 cm lateral to the midline, Figure 3A).
  b. Using your non-dominant hand, gently lift the skin to form a ‘tent’ (Figure 3B).
  c. Position the needle with the bevel facing up. Insert the needle approximately 0.5 cm into the flat side of the ‘tent’.
  d. Slowly dispense the full volume, ensure that a visible bulge (Figure 3C) is formed.
  e. Withdraw the needle in a straight motion and immediately dispose of it in a designed sharps container.

***Note:*** To prevent leakage or accidental spilling, avoid inserting the needle at an angle greater than 45 degrees. Ensure the injection site is located on the lower dorsal region, at a distance from the hindlimbs to prevent discomfort. In some cases, the injected suspension may travel toward the lower extremities, resulting in abscess formation near the hind limbs. Based on our previous experience, this does not negatively impact the well-being of the mice. Animals remain active, can climb enrichment objects, and move freely within the cage. We have not observed any instances of impaired mobility or inability to walk due to abscess location.

***Note:*** Injection placement closer to the hind leg or shaved fur boundary should be avoided, as this could affect lesion size measurements.

17. Use a permanent marker to outline the injection site on the skin (Figure 3D). The injected volume should appear as a slightly raised subcutaneous ‘bubble’, which will dissipate shortly after administration. Marking the site helps to reliably identify the original injection area for subsequent assessments or treatment.

***Note:*** If skin appears pale or white immediately after injection, this may indicate subdermal rather than subcutaneous delivery, which can lead to localized tissue necrosis.

18. Remove the mouse from anesthesia and keep it in a pre-warmed recovery chamber. Monitor the animal until it regains mobility and can move independently. Once fully recovered, return it to its group housing cage.

**CRITICAL:** Prepare all bacterial injections in advance, ideally grouped per cage, to reduce variability and animal exposure to isoflurane. Before each injection, gently mix the syringe to maintain a homogeneous suspension. Avoid vigorous shaking or bubble formation.

19. Inject therapeutic agents subcutaneously at the infection site 1–3 h after bacterial challenge.
  ***Note:*** Based on our previous studies, 1 h post-infection is the optimal time point for initiating treatment. This ensures early intervention while still allowing sufficient time for initial host–pathogen interaction.
  ***Optional:*** Delayed administration of therapeutic agents (up to 24 h) is feasible; however, once fluid accumulation or necrosis sets in, injection precision and drug distribution may be compromised.
  **CRITICAL:** Pre-test all therapeutic compounds for dermal toxicity (Figure 4).
  a. Prior to infection studies, shave and depilate the dorsal skin of mice and inject the compound at the intended dose.
  b. Test compounds at concentrations above the therapeutic range to evaluate safety and local tolerance.
  c. Also test vehicle controls (e.g., DMSO) at the exact concentrations to be used, as solvents alone may cause irritation, inflammation, or tissue damage.
    ***Note:*** DMSO is given here as an example. Different solvents may have varying effects on skin integrity. For DMSO specifically, concentrations above 10% can compromise skin barrier integrity by dissolving lipids in the epidermis, potentially causing mild inflammation, edema, and altered skin morphology.^17^^,^^18^
    **CRITICAL:** Avoid exceeding concentrations, as this may confound lesion appearance or immune readouts.
    ***Note:*** Alternatively, treatments can be administered intravenously (IV). In IV toxicity testing, 100 μL of the test compound at varying concentrations is injected via the tail vein. Mice are continuously monitored for signs of discomfort or adverse reactions following administration. If signs of toxicity are observed, animals should be placed on low heat and closely monitored during recovery. However, if they fail to recover within a few minutes or exhibit persistent signs of pain or distress, they must be euthanized immediately in accordance with humane endpoint criteria.
    ***Note:*** In our experience, skin toxicity is strongly correlated with intravenous (IV) toxicity. Compounds that induce visible tissue necrosis when administered subcutaneously (Figure 5) can cause significant systemic toxicity when delivered intravenously. Toxic concentrations often result in severe animal distress and may lead to mortality.

### Monitoring mouse welfare

**Timing: 30 min per day; once or twice daily (bacterial strain dependent)**

This section provides guidance for daily assessment of mouse welfare and disease progression following subcutaneous infection. It outlines standardized scoring of activity, appearance, hydration, pain, and abscess development to ensure consistent evaluation of animal health. Live imaging using IVIS allows non-invasive, real-time monitoring of bacterial burden and spatial localization, enabling dynamic assessment of infection progression and treatment efficacy. Following this protocol ensures reliable data collection while minimizing stress and maintaining animal welfare.

**Figure 6:**
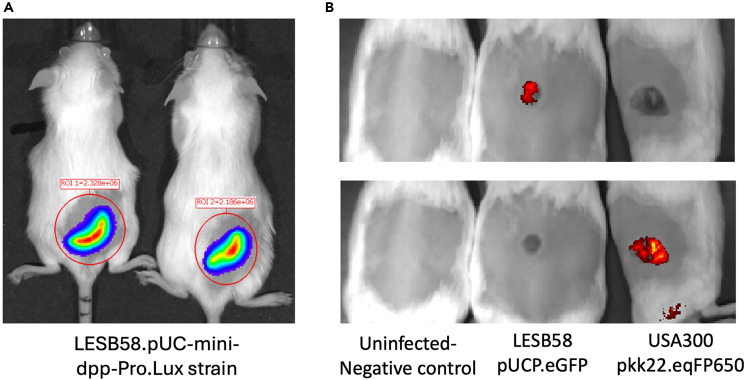
In vivo imaging of subcutaneous bacterial infections using the IVIS Spectrum system (A) Representative bioluminescence image of two mice infected with bioluminescent-labelled *Pseudomonas aeruginosa* LESB58. Signal indicates bacterial burden at the site of subcutaneous infection three days post infection, captured using the IVIS imaging system. (B) Fluorescence imaging of three mice: control (left), *P. aeruginosa* LESB58 tagged with eGFP (middle), and *S. aureus* USA300 expressing eqFP650 (right). Top panel was imaged with GFP filter settings, while bottom panel used eqFP650 filter settings (Table 3). Distinct fluorescent signals allow spatial differentiation of each pathogen during infection, visualized non-invasively using the IVIS system.

**Table 3:** Recommended IVIS imaging (living Image 4.8) settings for fluorescent and bioluminescent imaging

Step	Parameter	Fluorescence imaging	Bioluminescence imaging
1	Imaging mode	Select Fluorescence imaging mode	Select Bioluminescence imaging mode
2	Filter settings	Choose Ex/Em based on fluorophore:– GFP: Ex 465 nm/Em 520 nm– eqFP650: Ex 570 nm/Em 620 nm	No filters required
3	Binning	Set to Medium or High for sensitivity	Set to Medium or High depending on signal
4	Exposure time	Start with 1–5 s; optimize based on fluorescence intensity	Start with 1–60 s; adjust for signal strength
5	Field of view (FOV)	Adjust to capture entire animal or ROI	Adjust to capture entire animal or ROI
6	Imaging sequence	Image each fluorophore sequentially to avoid spectral overlap	Single acquisition; ensure consistent timing post-substrate injection
7	Substrate	Not applicable	Inject luciferin prior to imaging (timing per protocol)

#### Day 1 onwards: Monitoring

20. Monitor the disease progression and the welfare of the mice daily.
  a. Assess mouse activity, behavior, and hunching.
  b. Evaluate overall appearance.
  c. Check hydration status.
  d. Assess signs of pain.
  e. Examine abscess: note color (white, red, or dark-red) and presence of scabbing.
  f. Score mice using the developed chart (Data S1 and S2) to document welfare and abscess progression.
  g. Include standard monitoring such as BAR (bright, alert, reactive).

***Note:*** Monitoring may be required twice daily for untested bacteria and/or particularly aggressive infections.

***Note:*** Through ongoing consultation with veterinarians, we have determined that body weight is a poor metric for assessing animal well-being in this short-term infection model and may result in unnecessary handling and stress to the animals. Any concerning weight loss (e.g., >20%) would typically be accompanied by other observable clinical signs, such as changes in body condition, posture, or pain-related behaviors, which are already part of routine monitoring.

21. Real-time disease progression during subcutaneous infection using Live Imaging with the IVIS Spectrum system.
  ***Note:*** In Vivo Imaging Systems (IVIS) offer a powerful, non-invasive approach to monitor disease progression and microbial dynamics in real time within live animals. This technique relies on the detection of bioluminescent (Figure 6A) or fluorescent signals (Figure 6B) emitted by tagged pathogens or cells, enabling longitudinal studies without the need to euthanize animals at multiple time points. In the context of subcutaneous infection models, IVIS imaging allows for quantitative assessment of bacterial burden, spatial localization of infection, and treatment efficacy over time, providing a dynamic view of host-pathogen interactions in the skin.
  ***Note:*** Confirm bacterial bioluminescence or fluorescence (e.g., via plate reader, microscope or IVIS) prior infection studies.
  a. Prepare the mice and bacterial injection as mentioned above in step 1–13.
  b. Pre-Imaging Preparation.
    i. Anesthetize the mouse using isoflurane (e.g., 2–3% for induction, 1–2% for maintenance).
      ***Note:*** During daily anesthesia for IVIS imaging, mice are anesthetized using 2%–3% isoflurane in oxygen, following standard protocols (Table 1). Anesthesia sessions should be minimized, as prolonged or repeated isoflurane exposure has been associated with neurotoxicity and other adverse effects in rodents.^19^^,^^20^
    ii. Place the mouse in a prone position in the IVIS imaging chamber.
    iii. Ensure the region of interest (ROI) is exposed and flat (avoid folding of the skin).
  c. Setting Up the IVIS Parameters using Table 3 (for PerkinElmer devices).
    i. Check auto-adjusted scale or use a consistent scale for comparative studies.
  d. Acquire the Image.
    i. Adjust settings (exposure, binning, filters) if signal is too weak/strong.
  e. After imaging, remove the mice from the chamber and allow to recover over heating pad until fully awake.
  f. Return to cage and monitor until normal behavior resumes.
  g. Data Analysis.
    i. In the Living Image software (PerkinElmer), use ROI tools to quantify signal intensity (e.g., total radiant efficiency, average radiance).
    ii. Correct the *in vivo* radiance values for tissue attenuation by applying an experimentally determined skin attenuation factor (F). Experimentally determined F values for each fluorophore used in this study are summarized in Table S1, and all radiance values should be corrected using F before analysis.
      ***Note:*** F is calculated by imaging the same fluorescent bacterial suspension *in vitro* (R_free_) and in vivo (R_*in vivo*_) under identical IVIS settings and taking the ratio F = Rf_ree_ / R_in vivo_. All radiance values are multiplied by F before quantification. To ensure accurate and reproducible measurements, all *in vivo* experiments must be performed under consistent conditions, including animal strain, age, gender, and route of administration, as these factors can significantly affect fluorescence readings.
      ***Note:*** While using multiple fluorophores (e.g., eGFP, eqFP650), determine F separately for each fluorophore. Image multiple fluorophores sequentially to avoid bleed-through and confirm spectral separation with *in vitro* reference samples before *in vivo* experiments.
  h. Export quantitative data and representative images for documentation.
    ***Note:*** Always image control animals to correct for background autofluorescence. Use consistent imaging settings across time points and animals for reliable comparison. If imaging multiple fluorophores, image sequentially to avoid bleed-through.
    **CRITICAL:** Growing GFP- and RFP-expressing bacteria on agar plates and using them to optimize spectral unmixing on the IVIS system is a critical preliminary step. It is important to select fluorescent reporters with minimal spectral overlap and validate their compatibility before initiating *in vivo* experiments.
    **CRITICAL:** Accurate determination of the tissue attenuation factor (F) is essential for reproducible quantification. Without correction, radiance underestimates bacterial load due to skin absorption and scattering. Report mean F ± SD (or 95% CI) based on replicate measurements.

### Experimental endpoint and tissue processing

**Timing: ∼20 min per cage of 5 mice**

This section provides procedures for euthanizing mice, collecting and dissecting abscess tissue, and preparing samples for bacterial enumeration. It ensures accurate lesion measurement, standardized tissue homogenization, and reliable assessment of infection and treatment outcomes while maintaining animal welfare and minimizing contamination.

**Figure 7:**
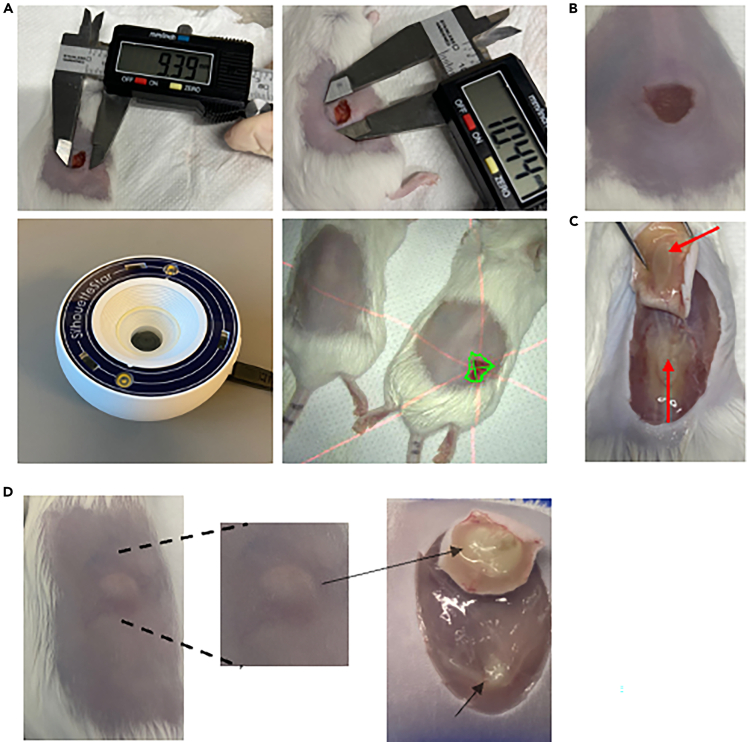
Abscess visualization and assessment of infection progression in mouse subcutaneous models (A) Measurement of abscess size using digital calipers (left, middle) or by using SilhouetteStar 3D wound camera (right) for accurate quantification. (B) Well-defined abscess visible on properly shaved skin. (C) Pus accumulation beneath the abscessed skin, indicating active infection. (D) Subclinical abscess with unclear external signs, yet bacterial pus is present beneath the skin surface.

#### Day 3 or at the endpoint of the experiment

22. Anesthetize mice to a surgical plane of anesthesia using 3%–5% isoflurane.
  a. Confirm deep anesthesia by performing a toe-pinch reflex test; the mouse should not respond.
  b. Perform cervical dislocation to ensure death, in accordance with approved ethical guidelines.
23. Disinfect the skin by wiping the area with 70% ethanol to reduce contamination from the natural skin microflora and minimize the risk of sample contamination.
24. Photograph mice.
25. Measure abscess lesion sizes (visible dermonecrosis) using a digital calliper (Marathon, CO030150), excluding swelling/inflammation from the measurements (Figure 7A, top). Alternatively, a wound camera (Figure 7A, bottom) can be used (e.g., Silhouette 3D wound camera).

***Note:*** Abscess size is measured based on visible lesion formation; surrounding swelling or inflammation is not included in the measurement but recorded separately as observational data. The wound camera has certain limitations, such as its inability to measure raised tissue or lumps accurately; however, it performs well in capturing and assessing visible surface wounds.

***Note:*** At this stage, other organs can also be dissected from the mouse and stored for downstream analysis.

26. Carefully dissect the abscess (Figures 7B and 7C) from the mouse and place the tissue into a microcentrifuge tube.
  a. After transferring the tissue to the collection tube, cut the abscess into smaller pieces using sterile dissecting scissors to facilitate homogenization.
    ***Note:*** Tissue isolation should be performed in sterile conditions. Keep dissecting instruments in 200 mL of 70% EtOH between uses.
  b. During dissection, take care to scrape any pus or exudate from the underlying tissue using sterile forceps, as this often contains viable bacteria and is important for accurate bacterial load quantification (Figure 7C).
  c. If antimicrobial treatment has been effective, abscess formation may be absent. In such cases, use the initial skin markings to identify the injection site, then carefully dissect the overlying skin from the body to expose the subdermal space and inspect for residual signs of infection. (Figure 7D).
27. Add sterile PBS to each microcentrifuge tube up to 1 mL. Add 3–5 sterile ceramic beads (Qiagen) to each tube and homogenize using e.g., a tissue lyser at 2,500 rpm for 5 min (MiniG 1600 Specs Sample prep) or Qiagen Tissuelyser III, 2-3 min with 25 Hz.

***Note:*** This protocol uses a total volume of 1 mL PBS to maximize bacterial recovery from the full abscess area regardless of tissue size. Researchers who prefer normalization by tissue mass may weigh the dissected tissue and homogenize it at a defined weight-to-volume ratio (e.g., 100 mg tissue per 1 mL PBS) to allow for standardized comparisons across samples.

***Note:*** We recommend using sterile ceramic beads for homogenizing skin tissue instead of glass beads. Ceramic beads are more durable and provide more efficient mechanical disruption of dense or fibrous tissues like skin, resulting in better homogenization.^21^ Glass beads can shatter under high-speed agitation, which poses a safety risk and potentially contaminates samples with glass fragments. However, glass beads are well suited for downstream processes such as lysis of microorganisms.

***Note:*** Use the recommended time and speed with the type of tissue lyser being used, for e.g., with Qiagen Tissuelyser III, 2–3 min with 25 Hz.

28. Transfer 20 μL from each tube and serially dilute in a 96-well plate.
29. Spot 10 μL from each well onto individual quadrants of a nutrient-rich agar plate.
  Spread the spot in each quadrant.

***Note:*** Alternatively, samples can be plated onto selective media to differentiate bacterial species in polymicrobial infections (e.g., *P. aeruginosa* on cetrimide agar, *S. aureus* on 7.5% NaCl agar).

30. Incubate plates overnight at 37°C for 12–16 h.

#### Day 4

31. Plates from day 3 can be counted for bacterial enumeration.

### Downstream processes following murine subcutaneous skin infection

Following tissue collection and homogenization, host immune responses and bacterial burden in skin infections can be further evaluated using a variety of methods. The downstream applications outlined below highlight the versatility and suitability of this abscess model for diverse experimental approaches. Assay selection depends on the specific study objectives but commonly includes CFU enumeration to assess bacterial load, histology, flow cytometry for immune cell profiling, and RNA extraction for transcriptomic analysis. Table 4 below summarizes functional assays that can be easily used with this skin abscess model.

**Table 4:** Representative downstream analyses that can be performed using samples collected from the murine abscess model

Process	Application	Method in brief	Readouts
Bacterial quantification	Colony forming unit (CFU) assay	Homogenize abscess tissue in PBS, perform serial dilutions, and plate on selective media	Bacterial load (CFU per abscess or per gram tissue)
Bacterial transcriptomics	Bacterial RNA-seq/qRT-PCR	Isolate bacterial RNA from abscess tissue using enrichment kits, prepare libraries for sequencing or qRT-PCR	Differential gene expression, virulence factor expression
Host transcriptomics	Host RNA-seq/qRT-PCR	Extract total RNA from tissue around the infection site, deplete rRNA or enrich mRNA via poly(A) selection, prepare libraries or use cDNA for qRT-PCR	Host immune gene signatures, inflammatory pathways
Genomics	Bacterial whole-genome sequencing/Tn-seq	Isolate genomic DNA from bacteria within abscess; prepare libraries for WGS or perform Tn-seq to study mutant fitness	Mutation profiling, bacterial fitness during infection
Proteomics	Host or bacterial protein analysis	Isolate proteins from infected tissue; perform Western blot, Mass spectrometry, or proteome profiling	Protein expression, cytokines, virulence factors
Metabolomics	Host/bacterial metabolic state	Extract metabolites from abscess using methanol or chloroform extraction; analyze via LC-MS or GC-MS	Metabolic shifts during infection, nutrient availability
Immune profiling	Flow cytometry/FACS	Digest tissue into single-cell suspension, stain with antibodies for immune subsets	Immune cell infiltration, activation markers
Cytokine profiling	ELISA, LEGENDplex, Luminex, MSD	Use tissue supernatants or homogenates; detect cytokines using sandwich ELISA, multiplex bead-based LEGENDplex, Luminex or electrochemiluminescence MSD platform	Quantitative cytokine levels, multiplex cytokine profiling
Histopathology	Hematoxylin and Eosin (H&E), Gram, and immunohistoche-mistry (IHC) staining	Fix tissue in formalin or paraformaldehyde, embed, section, stain with H&E, Gram or antibodies	Tissue architecture, bacterial localization, immune infiltration
Bacterial live imaging	IVIS/Fluorescence microscopy	Infect mice with luminescent/fluorescent bacteria	Bacterial spread, localization
Host live imaging	IVIS/Fluorescence microscopy	Image infected mice using bioluminescence or near-infrared probes (e.g., detect reactive oxygen species, neutrophil markers, metalloprotease markers)	Immune response; spatial immune response
Drug live imaging	IVIS/Fluorescence microscopy	Drugs can be attached to luciferase or fluorescent probes	Drug distribution; pharmacokinetics
Drug efficacy testing	Antibiotic or compound screening	Treat mice post-infection and assess outcomes through lesion size, CFUs, or survival	Reduction in bacterial load, lesion resolution
Other Functional assays	Phagocytosis, ROS, bacterial killing	Tissue-derived leukocytes can be isolated and subjected to ex vivo assay for phagocytosis (e.g., using fluorescently labeled bacteria or beads), bacteria killing by quantifying CFUs, and ROS production (via DCFDA or luminol-based assays).	%phagocytic cells, killing efficiency (CFU counts), ROS intensity

## Expected outcomes

This protocol establishes an *in vivo* subcutaneous infection model in mice to study infections caused by Gram-positive, Gram-negative or polymicrobial (mixed) pathogens. Within 24–48 h post-infection, mice consistently develop localized abscesses that mimic key features of human soft tissue infections. Lesion progression can be monitored non-invasively through visual inspection or imaging platforms, and infected tissues harvested on Day 3 typically yield high and quantifiable bacterial loads (CFUs).

Therapeutic interventions are expected to reduce both lesion size and bacterial burden, offering a tractable platform for evaluating treatment efficacy and studying immune responses *in vivo*. This model is particularly suited for testing antimicrobial agents under physiologically relevant, high-density infection conditions.

Given that more than two-thirds of hospital-acquired infections involve chronic or biofilm-related pathogens that are difficult to eradicate due to adaptive multidrug resistance, this model provides a simplified yet powerful tool to address a significant unmet clinical need. It overcomes limitations of traditional chronic infection models by enabling longitudinal infection tracking without the need for repeated animal sacrifice.

## Limitations

Abscess formation in this model can be influenced by several factors, including mouse age, genetic strain, and batch-to-batch variability in bacterial virulence. To minimize variation, it is essential to use bacteria at a consistent growth phase and ensure proper shaving technique. Skin that is burnt or abraded during preparation may elicit exaggerated immune responses, potentially confounding infection dynamics.

Therapeutic compounds are administered as early as 1 h post-infection in this model, and we acknowledge that such timing does not reflect typical clinical scenarios. This protocol is not designed to directly mimic human skin infections, but rather to provide a reproducible *in vivo* platform for studying infection progression and evaluating treatment efficacy under controlled, high-density infection conditions. When establishing this model, we found that treatment administered at 1, 3, or even 24 h post-infection yielded comparable outcomes, supporting its flexibility for testing therapeutic interventions at different stages of infection.

## Troubleshooting

### Problem 1

Incomplete hair removal after the depilatory step.

### Potential solution

Insufficient exposure time or uneven application of depilatory cream may cause incomplete hair removal. Rinse the depilatory thoroughly, dry the area, and reapply a fresh layer for exactly 1 min only. Do not extend exposure beyond 2 min, as longer application can cause burns or skin irritation. Refer to Step 2, c. i-viii (*Application of chemical depilatories and removal)*.

### Problem 2

Hair regrowth occurs rapidly (within 1 day) after shaving, potentially obscuring lesion development or interfering with imaging and assessment.

### Potential solution

While shaving several days before infection is acceptable, it requires close monitoring-especially in young mice (<5 weeks old), where visible hair regrowth can occur within 2–3 days. To maintain a clear infection site, re-trim or reapply depilatory cream shortly before infection if necessary. Avoid using very young (<5 weeks) or older mice (<8 weeks); younger mice exhibit faster fur regrowth, which may hinder abscess formation, while older mice typically have thicker skin, resulting in smaller lesions (see Figure 2F).

### Problem 3

Variability in inoculum preparation can result in inconsistent CFU delivery, affecting lesion development and experimental reproducibility.

### Potential solution

To minimize variability, thoroughly vortex the bacterial suspension before aliquoting and shake the injection syringe immediately before each injection to prevent settling. Use consistent pipetting techniques to deliver accurate volumes, and standardize bacterial culture conditions and optical density measurements to normalize CFU. Prepare fresh inoculum for each experiment and, verify CFU by plating a small aliquot of each batch before injection. Refer to Step 5 (bacterial inoculum preparation on Day 0).

### Problem 4

Mis-injection of the bacterial inoculum (e.g., intradermal or intramuscular instead of subcutaneous).

### Potential solution

Ensure needle insertion is subcutaneous at 1–2 mm depth, parallel to the skin surface. Refer to Step 16 (subcutaneous infection and treatment) and Figure 3A.

### Problem 5

A mouse develops an open wound at the infection site or shows signs of excessive lesion aggravation.

### Potential solution

In rare cases, lesions may become aggravated (scratching, bleeding), which deviates from the typical presentation of a localized, subcutaneous lump without overt pain or open wounds. Such responses may result from external factors, including sharp cage objects, aggressive cage mates, or excessive grooming.

If lesion aggravation occurs, immediately remove the affected animal and euthanize in accordance with humane endpoint guidelines. To prevent recurrence, inspect cage environments regularly for hazards and monitor mouse behavior closely for early signs of distress or social aggression. Also, refer to Step 20 (monitoring mouse welfare).

### Problem 6

A mouse shows signs of self-inflicted trauma (e.g., scratching leading to broken skin) at the injection site, which meets humane endpoint criteria.

### Potential solution

While mild irritation at the inoculation site is expected, animal welfare should be supported by ensuring adequate hydration (Step 20, monitoring mouse welfare). Monitor mice closely for signs of distress or excessive grooming. If trauma occurs, euthanize the animal immediately following ethical guidelines. Based on prior experience with this model, such cases are rare and typically do not occur under standard housing and experimental conditions.

## Resource availability

### Lead contact

Further information and requests for resources and reagents should be directed to and will be fulfilled by the lead contact, Daniel Pletzer (daniel.pletzer@otago.ac.nz).

### Technical contact

Questions about the technical specifics related to performing the protocol should be directed to and answered by the technical contact, Anupriya Gupta (anupriya.gupta@otago.ac.nz).

### Materials availability

This study did not generate new, unique reagents.

### Data and code availability

This study did not generate any data or code.

## Acknowledgments

D.P. and A.G. acknowledge funding from the 10.13039/501100009193Royal Society of New Zealand Marsden Fund (MFP-UOO2203). K.J.S. acknowledges support from the 10.13039/100018641University of Otago Doctoral Scholarship. J.D.S. was supported by a Te Niwha MSc Scholarship (TN/PLSM/24/48/UOOJS and AP41789_CON26674). D.P. and S.J.T.W. also acknowledge funding from the 10.13039/100018641University of Otago Research Grant.

We are grateful to Nikita Lyons and Shuying Wee for their contributions to the initial drafts of the protocol. We also thank the staff of the Eccles animal facility, particularly the technicians Nicky McConnachie, Louise Belsham, Joanne Fissenden, and Tash Renshaw, for their assistance with animal preparation and shaving.

## Author contributions

D.P. and A.G.: conceptualization; D.P., A.G., K.J.S., and J.D.S.: methodology, validation, and investigation; A.G. and K.J.S.: writing – original draft; A.G., S.J.T.W., and D.P.: review and editing; A.G. and D.P.: figure preparation; D.P.: supervision and funding acquisition.

## Declaration of interests

The authors declare no competing interests.

## Supplemental information

Document S1. Figure S1 and Table S1

Data S1. Scoring chart for monitoring mouse welfare and abscess progression, related to Step 20 A structured chart used to assess key indicators of mouse health following subcutaneous infection. The chart includes five key parameters: activity and behaviour, appearance, hydration status, pain indicators, and abscess severity. Each parameter is scored from 0 to 3, with 0 indicating normal condition and 3 indicating severe distress or pathology. Any mouse receiving a grade 3 in any single category is considered to have reached humane endpoint and should immediately be euthanized.

Data S2. Monitoring sheet, related to Step 20

## References

[1] Pletzer D., Mansour S.C., Wuerth K., Rahanjam N., Hancock R.E.W. (2017). New Mouse Model for Chronic Infections by Gram-Negative Bacteria Enabling the Study of Anti-Infective Efficacy and Host-Microbe Interactions. mBio.

[2] Pletzer D., Mansour S.C., Hancock R.E.W. (2018). Synergy between conventional antibiotics and anti-biofilm peptides in a murine, sub-cutaneous abscess model caused by recalcitrant ESKAPE pathogens. PLoS Pathog..

[3] Wardell S.J.T., Yung D.B.Y., Gupta A., Bostina M., Overhage J., Hancock R.E.W., Pletzer D. (2025). DJK-5, an anti-biofilm peptide, increases Staphylococcus aureus sensitivity to colistin killing in co-biofilms with Pseudomonas aeruginosa. NPJ Biofilms Microbiomes.

[4] Wardell S.J.T., Yung D.B.Y., Nielsen J.E., Lamichhane R., Sørensen K., Molchanova N., Herlan C., Lin J.S., Bräse S., Wise L.M. (2025). A biofilm-targeting lipo-peptoid to treat Pseudomonas aeruginosa and Staphylococcus aureus co-infections. Biofilm.

[5] Pletzer D., Wolfmeier H., Bains M., Hancock R.E.W. (2017). Synthetic Peptides to Target Stringent Response-Controlled Virulence in a Pseudomonas aeruginosa Murine Cutaneous Infection Model. Front. Microbiol..

[6] Nielsen J.E., Alford M.A., Yung D.B.Y., Molchanova N., Fortkort J.A., Lin J.S., Diamond G., Hancock R.E.W., Jenssen H., Pletzer D. (2022). Self-Assembly of Antimicrobial Peptoids Impacts Their Biological Effects on ESKAPE Bacterial Pathogens. ACS Infect. Dis..

[7] Kłodzińska S.N., Pletzer D., Rahanjam N., Rades T., Hancock R.E.W., Nielsen H.M. (2019). Hyaluronic acid-based nanogels improve in vivo compatibility of the anti-biofilm peptide DJK-5. Nanomedicine..

[8] Wolfmeier H., Wardell S.J.T., Liu L.T., Falsafi R., Draeger A., Babiychuk E.B., Pletzer D., Hancock R.E.W. (2022). Targeting the Pseudomonas aeruginosa Virulence Factor Phospholipase C With Engineered Liposomes. Front. Microbiol..

[9] Rajchakit U., Lamba S., Wang K., Lyons N., Lu J., Swift S., Pletzer D., Sarojini V. (2024). Size-Controlled Synthesis of Gold Nanoparticles Tethering Antimicrobial Peptides with Potent Broad-Spectrum Antimicrobial and Antibiofilm Activities. Mol. Pharm..

[10] Lyons N., Wu W., Jin Y., Lamont I.L., Pletzer D. (2024). Using host-mimicking conditions and a murine cutaneous abscess model to identify synergistic antibiotic combinations effective against Pseudomonas aeruginosa. Front. Cell. Infect. Microbiol..

[11] Centers for Disease Control and Prevention (CDC) (2003). Outbreaks of community-associated methicillin-resistant Staphylococcus aureus skin infections--Los Angeles County, California, 2002-2003. MMWR Morb. Mortal. Wkly. Rep..

[12] Cheng K., Smyth R.L., Govan J.R., Doherty C., Winstanley C., Denning N., Heaf D.P., van Saene H., Hart C.A. (1996). Spread of beta-lactam-resistant Pseudomonas aeruginosa in a cystic fibrosis clinic. Lancet.

[13] Pletzer D., Blimkie T.M., Wolfmeier H., Li Y., Baghela A., Lee A.H.Y., Falsafi R., Hancock R.E.W. (2020). The Stringent Stress Response Controls Proteases and Global Regulators under Optimal Growth Conditions in Pseudomonas aeruginosa. mSystems.

[14] Wu B.C., Haney E.F., Akhoundsadegh N., Pletzer D., Trimble M.J., Adriaans A.E., Nibbering P.H., Hancock R.E.W. (2021). Human organoid biofilm model for assessing antibiofilm activity of novel agents. NPJ Biofilms Microbiomes.

[15] Sezonov G., Joseleau-Petit D., D'Ari R. (2007). Escherichia coli Physiology in Luria-Bertani Broth. J. Bacteriol..

[16] Navarro Llorens J.M., Tormo A., Martínez-García E. (2010). Stationary phase in gram-negative bacteria. FEMS Microbiol. Rev..

[17] Guo W., Qiu W., Ao X., Li W., He X., Ao L., Hu X., Li Z., Zhu M., Luo D. (2020). Low-concentration DMSO accelerates skin wound healing by Akt/mTOR-mediated cell proliferation and migration in diabetic mice. Br. J. Pharmacol..

[18] Senna T.D., Mata Dos Santos H.A., Kibwila D.M., Leitao A.C., Santos Pyrrho A.D., de Padula M., Rosas E.C., Padua T.A., Lara M.G., Riemma Pierre M.B. (2017). In Vitro and In Vivo Evaluation of DMSO and Azone as Penetration Enhancers for Cutaneous Application of Celecoxib. Curr. Drug Deliv..

[19] Wilding L.A., Uchihashi M., Bergin I.L., Nowland M.H. (2015). Enucleation for treating rodent ocular disease. J. Am. Assoc. Lab. Anim. Sci..

[20] Jevtovic-Todorovic V., Hartman R.E., Izumi Y., Benshoff N.D., Dikranian K., Zorumski C.F., Olney J.W., Wozniak D.F. (2003). Early Exposure to Common Anesthetic Agents Causes Widespread Neurodegeneration in the Developing Rat Brain and Persistent Learning Deficits. J. Neurosci..

[21] Yu V.M., Micic M. (2016). Sample Preparation Techniques for Soil, Plant, and Animal Samples.

