# Supplementary material for: Protocol for a murine skin abscess model to study bacterial infection dynamics, microbial interactions, and treatment efficacy
Source: STAR Protoc. 2025 Dec 20;7(1):104291. doi: 10.1016/j.xpro.2025.104291 (PMC12794494; doi:10.1016/j.xpro.2025.104291)
Supplement: Document S1. Figure S1 and Table S1 [file mmc1.pdf]

Supplemental Figure

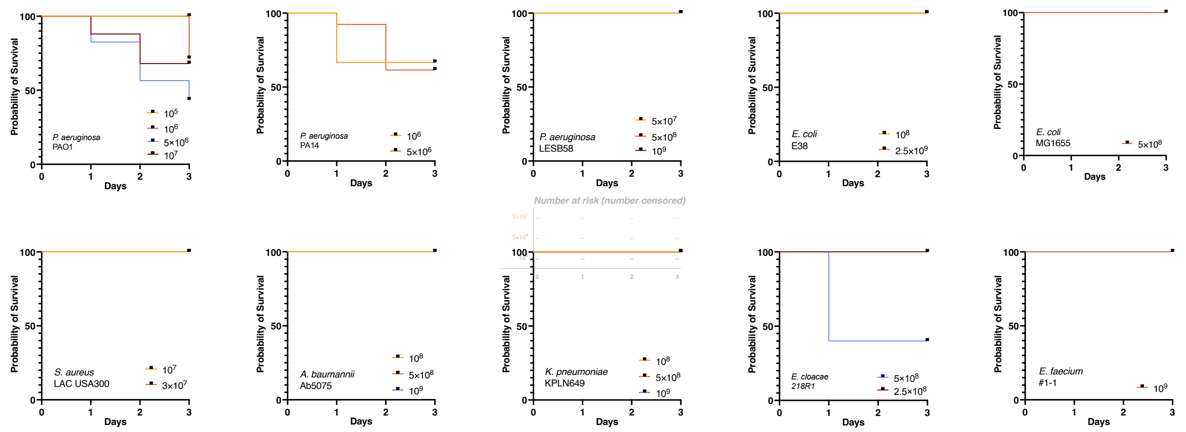

Supplementary Figure S1: Survival analysis of murine subcutaneous infection model, related to section ‘Before you begin’. Kaplan-Meier survival curves for mice inoculated subcutaneously with increasing doses of representative bacterial pathogens. Strains include *P. aeruginosa* PAO1 (n = 5, 7, 23, 25), PA14 (n = 3, 13), LESB58 (n = 14, 4, 4); *E. coli* E38 (n = 11, 9), MG1655 (n = 6); *S. aureus* USA300 (n = 6, 10); *A. baumannii* Ab5075 (n = 3, 3, 10); *K. pneumoniae* KPLN649 (n = 3, 9, 7); *E. cloacae* 128R1 (n = 5, 9, 5); and *E. faecium* #1-1 (n = 12). All mice survived the 3-day experimental window, confirming that the model yields localized, non-lethal infections under the tested conditions.

Supplemental Table

Supplementary Table S1: Experimentally determined tissue attenuation factors (F) for fluorescent reporters used in murine cutaneous infection models, related to Step 21. Attenuation factors (F) were experimentally determined by measuring the radiance of bacterial suspensions in vitro (R_free) and in vivo (R_in vivo) under identical IVIS settings. F represents the fold correction applied to in vivo radiance values to account for tissue absorption and scattering. All quantitative imaging done in this protocol is corrected using these F values.

Fluorophore Excitation/ Emission (nm)	Strain	Mean R_free (a.u.)	Mean R_invivo (a.u.)	Attenuation factor F (R_free / R_in vivo)	Attenuation strength
**eGFP** 488/510	*P. aeruginosa* LESB58	1.78×10^8	2.09×10^9	**0.14 ± 0.02**	Strong
**eqFP650** 592/650	*S. aureus* USA300	4.81×10^7	4.38×10^9	**0.35 ± 0.05**	Moderate
